# Supplementary material for: Hypertensive crisis: Insights into prevalence and associated factors at a tertiary care facility in Zambia
Source: PLOS Glob Public Health. 2025 May 20;5(5):e0004649. doi: 10.1371/journal.pgph.0004649 (PMC12091803; doi:10.1371/journal.pgph.0004649)
Supplement: Attachment — Submitted filename: reviewer responses.docx [file pgph.0004649.s004.docx]

**Review Comments to the Author**

We want to thank the reviewers for their invaluable suggestions that have improved our manuscript. We have revised accordingly and below are the specific responses to reviewer comments:

Reviewer #1: Significance, Novelty, and Strengths.

The paper addresses a critical health issue, the prevalence of hypertensive crisis, and its associated factors in a significant population at a referral center in Zambia. This is an important contribution to the understanding of hypertension in developing countries, where such data might be scarce.

The study’s originality is demonstrated through its focus on a specific population in Zambia and the use of robust data collection methods from a large sample size (n=977). Additionally, the paper’s approach to using both univariate and multivariable logistic regression analysis to identify factors associated with hypertensive crisis adds depth to the findings.

Methodology: The retrospective cross-sectional design is well-suited to the research question. The systematic sampling method used to select patient records and the clear inclusion and exclusion criteria bolster the study's validity.

Analysis: The use of REDCap for data capture and STATA for statistical analysis ensures robust data handling and analysis. The distinction between univariate and multivariable logistic regression provides comprehensive insights into the factors associated with hypertensive crisis.

Relevance: Given the high prevalence of hypertension in sub-Saharan Africa, this study is highly relevant to public health in this region. The findings have potential implications for clinical practice and policy-making in Zambia and similar contexts.

**Response:** thank you for providing an accurate appraisal of our manuscript.

Weaknesses:

Missing Data: The exclusion of 79 files due to missing data may introduce selection bias. The paper lacks a discussion on how this limitation might affect the study's findings.

Generalizability: The study is conducted in a single referral hospital, which may limit the generalizability of the results to other settings in Zambia or other countries.

Confounding Factors: While the study includes several variables, it does not address potential confounding factors such as diet, physical activity, and genetic predisposition that could influence hypertensive crisis.

Depth of Discussion: The discussion section could be more comprehensive, particularly in contextualizing the findings within the broader literature and discussing the implications for clinical practice and policy.

Suggestions for Improvement:

Address Missing Data: Provide a detailed analysis of the potential impact of the 79 excluded files on the study’s findings. Discuss any patterns in the missing data and use sensitivity analysis to understand how this exclusion might influence the results.

**Response:** thank you very much. We did the sensitivity analysis for the model before exclusion and after inclusion of all variables the results didn’t match. Compliance to medication, hypertensive status, employment status and hospitalization remained significant. Under the data analysis section we have explained the sensitivity analysis was done to account for exclude variables.

Enhance Generalizability: While acknowledging the limitations of a single-center study, suggest strategies for future research to include multiple centers across different regions to enhance the generalizability of the findings.

**Response:** thank you very much for your suggestion. We have now included in the discussion, the suggestion for future research strategies to enhance generalization of results.

Control for Confounders: Incorporate additional variables related to lifestyle and genetic factors in future analyses to control for potential confounding factors. If data on these variables were not available, discuss this limitation and its potential impact on the study's conclusions.

**Response:** thank you very much for your suggestion. We have included the suggestion for future research strategies to enhance generalization of results.

Expand Discussion: Strengthen the discussion section by comparing the study's findings with existing literature from similar contexts. Discuss the implications of the high prevalence of hypertensive crisis for healthcare providers and policymakers in Zambia. Provide more detailed recommendations for practice and policy based on the study’s findings.

**Response:** Thank you very much for your suggestion. We have strengthened the studies as suggested by providing more references in a similar context to ours and discussed the implication of the high prevalence of the hypertensive crisis for policymakers and health providers in Zambia. Line 198 to 209.

Ethical Considerations: Given the retrospective nature of the study, it would be beneficial to discuss how the ethical considerations were managed, particularly concerning patient confidentiality and data security.

**Response:** thank you very much. To ensure confidentiality we made sure that no identifying information such as particular hospital identification number, name or anything that can be used for identification was not abstracted. We have added on this information on the ethics section under methodology.

Reviewer #2: Areas for Improvement
Confounding Variables: While the multivariable logistic regression approach is appropriate, it is not clear which variables were included as potential confounders in the final model. Providing a rationale for the selection of independent variables or adjusting factors would enhance the transparency of the analysis.

**Response:** Thank you very much for your concern. To determine the variables we used in the final model, we selected and included variables based on the published literature and those variables that were significant in bivariable analysis. We have now added this information under data analysis method section line 119- 122.

Handling of Missing Data: There is no mention of how missing data, if any, were handled during the analysis. A description of missing data management (e.g., exclusion, imputation, or sensitivity analysis) would strengthen the methodological rigor.

**Response:** Thank you very much for your concern. We did the sensitivity analysis for the model before exclusion and after inclusion of all variables the results didn’t match. Compliance to medication, hypertensive status, employment status and hospitalization remained significant.

Model Assumptions: It is important to ensure that the assumptions of logistic regression were checked. Consider including diagnostic tests such as multicollinearity assessment (using Variance Inflation Factor) or goodness-of-fit tests (e.g., Hosmer-Lemeshow test).

**Response:** Thank you very. Please See responses above under confounding variable.

Clarity in Subgroup Analyses: Some subgroup analyses, such as those related to age and medication adherence, could benefit from clearer statistical justification. If p-values or confidence intervals are available for these comparisons, including them would help highlight the statistical significance of these findings.

**Response:** we have included the suggestion. Thank you

3. Methodological Concerns
Definition of Outcome: The primary outcome (hypertensive crisis) is well-defined; however, further clarification on how "target organ damage" was assessed by clinicians would be beneficial for reproducibility. Consider providing examples or criteria for target organ damage if available.

**Response: thank you. We have now provided examples on how TOD was assessed in our study.**

Detailed Description of Study Design: While the study is retrospective and cross-sectional, further details on the data extraction process and criteria for inclusion/exclusion would improve the transparency of the study design.

**Response: thank you. We have provided detail analysis on data extraction under eligibility and methods section as well as figure 1.**
