# Supplementary material for: Hypertensive crisis: Insights into prevalence and associated factors at a tertiary care facility in Zambia
Source: PLOS Glob Public Health. 2025 May 20;5(5):e0004649. doi: 10.1371/journal.pgph.0004649 (PMC12091803; doi:10.1371/journal.pgph.0004649)
Supplement: Attachment — Submitted filename: Response to the reviewer.docx [file pgph.0004649.s005.docx]

**Response to the reviewer**

**Reviewer #1**: Lines 31 and 168: In the results section, please revise the presentation of the confidence interval (CI) for the prevalence of hypertensive crisis. Currently, it is reported as:

"The prevalence of hypertensive crisis was 18.9% (95% CI: 0.17, 0.21)."

For better readability and consistency with standard epidemiological reporting formats, we recommend rephrasing it as:

"The prevalence of hypertensive crisis was 18.9% (95% CI: 17.0%, 21.0%)."

**Response:** thank you for the correction. We have corrected the phrase in line 31 and line 168 as suggested.

**Reviewer #2: Ambiguity in Findings:**
The high adjusted odds ratio for hypertension (AOR: 29.7; 95% CI: 2.30–384.1) raises concerns about wide confidence intervals, suggesting possible issues with data variability or sample size for this factor.

**Response:** thank you for the observation. We have reanalyzed removed it from the final model due to its wide confidences in analysis in hypertensive crisis and in the analysis of hypertensive components (emergency and urgency).

Distinguish Emergency vs. Urgency:
Analyze and report factors separately for hypertensive emergencies and urgencies, as their management and implications differ significantly.

**Response:** thank you for the suggestion. Indeed the management of HTN emergency and urgency differs significantly. We have analyze and reported the factors associated with HTN emergency and Urgency separately and added two more tables in the results section.
