# Supplementary material for: Hypertensive crisis: Insights into prevalence and associated factors at a tertiary care facility in Zambia
Source: PLOS Glob Public Health. 2025 May 20;5(5):e0004649. doi: 10.1371/journal.pgph.0004649 (PMC12091803; doi:10.1371/journal.pgph.0004649)
Supplement: Attachment — Submitted filename: Response to reviewers (1) (1).docx [file pgph.0004649.s006.docx]

**Response to reviewers**

Reviewer #1: The authors have made the necessary revisions based on our comments. The article has been significantly improved and now presents clear, coherent, and scientifically robust content. It is ready for publication and deserves to be accepted. No issues related to dual publication, research ethics, or publication ethics have been identified.

**Response:** Thank you.

Reviewer #2:

**Methods Section**

Insufficient Details:

1. The description of the retrospective cross-sectional design omits key elements, such as how participants were selected and whether records with missing data were excluded.

**Response:** thank you for the observation. Participants missing key information, such as the final diagnosis, were excluded from the final analysis, as shown in Figure 1 and described under “*Eligibility and sampling method”*. To select participants, we used a systematic sampling method, where every fifth file from the patient records in the department was selected for screening and eligibility, we have highlighted this under eligibility and method section (lines 113 to 120).

2. The methodology for identifying target organ damage is unclear. Was it based solely on the attending clinician’s judgment?

**Response:** Thank you very much. The clinician made the diagnosis using combinations of clinical diagnosis and investigations, as we explained in lines 108 to 109.

Potential Bias: The abstract does not address potential biases inherent in a retrospective design, such as data quality issues or missing information.

**Response:** Thank you for your observation. The clinical measurements were collected by trained healthcare professionals, and the hospital regularly conducts data audits to ensure data accuracy. Therefore, we assumed that the data collected was of high quality. Regarding the missing information in some variables, we have acknowledged this as a limitation of the study: 'Thirdly, the study may have been underpowered due to missing information in some variables' (lines 375–376).

3. The explanation of multivariable logistic regression is too brief. There’s no indication of how variables were selected for inclusion or how confounding was managed.

Handling of outliers (e.g., extremely high blood pressure values) is not mentioned.

**Response:** Thank you very much. The final model’s variables were chosen based on previously published literature and variables that were statistically significant at bivariable analysis; we highlighted this in lines 161 to 162. Outliers were checked for during the cleaning process and using the Shapiro-Wilk test for the quantitative data.

**Results Section**

Incompleteness:

4. While the prevalence is reported, the clinical or public health implications of hypertensive crisis prevalence are not explored.

**Response**: Thank you very much. To explore the public health implications, we have explored them in the discussion first paragraph .

5. The age and gender distribution are described but lack analysis. Why younger individuals were predominantly affected?

**Response**: Thank you very much. We have explored and given a detailed analysis of age and gender distribution in the discussion section from lines 280 to 290.

6. Overemphasis on Statistical Significance: The mention of p-values is necessary, but the abstract does not interpret the practical significance of the findings (e.g., how employment or hospitalization contributes to hypertensive crisis).

**Response**: thank you very much. In order to keep the abstract brief we opted to discuss the practical implication in the conclusion and discussion section.

7. Superficial Reporting: The distinction between hypertensive urgency (17.8%) and emergency (1.1%) is reported without elaboration on their clinical significance.

**Response**: thank you very much. To keep the abstract brief we opted to discuss the practical implications in the conclusion and discussion section.

Conclusion Section

8. Repetition: The conclusion repeats the results (e.g., prevalence and associated factors) instead of summarizing the broader implications for practice or policy.

**Response:** thank you, we have revised the section accordingly.

9. Generic Recommendations: While public awareness and medication adherence are emphasized, no specific strategies or next steps are provided, limiting the conclusion's utility.

**Response**: thank you for the recommendation. We have now highlighted specific strategies based on our findings which include community-based health education campaigns, which include outreach programs like mobile clinics, door-to-door campaigns, and digital outreach through social media, radio, and TV to raise awareness for hypertension; and patient-centered approaches, like integrating adherence counseling into routine care and leveraging digital health tools for reminders and follow-ups to improve adherence.

10. Overgeneralization: The abstract claims hypertensive crisis is a "significant proportion" without specifying what this means in a broader context, such as the burden on healthcare resources or patient outcomes.

**Response**: thank you for the recommendation. The broad context of the study has been highlighted in the conclusions and reduced over generalization of results.

General Issues

11. Wordiness: The abstract could be more concise by removing repetitive phrases and focusing on the most critical findings.

**Response**: thank you. We have revised the abstract to make it concise.

12. Clarity: Key terms such as "employment" and "hospitalization" are mentioned as risk factors but lack context. Why these variables are significant is not addressed.

**Response**: thank you. We have now endeavored to explore these variables and added details in the discussion section (see lines 288 and 297)

13. Engagement: The abstract does not effectively highlight the urgency or relevance of the findings to the healthcare system or public health.

**Response** thank you. We have revised the abstract to make it concise.

Suggestions for Improvement

14. Clearly articulate the study's significance in the background without referencing external comparisons.

**Response:** thank you. we have revised accordingly by removing external comparison of the problem of our study.

15. Provide more methodological transparency, including criteria for target organ damage and handling of missing or incomplete records.

**Response:** thank you. We have revised accordingly in the methods line 96 to 99.

Focus on the key findings without excessive repetition, ensuring that each section adds value.

**Response:** Thank you so much, the revision was made accordingly.

Strengthen the conclusion with actionable recommendations rather than merely reiterating results.

**Response**: Thank you for the advice, we revised the section accordingly.
