# Supplementary material for: Hypertensive crisis: Insights into prevalence and associated factors at a tertiary care facility in Zambia
Source: PLOS Glob Public Health. 2025 May 20;5(5):e0004649. doi: 10.1371/journal.pgph.0004649 (PMC12091803; doi:10.1371/journal.pgph.0004649)
Supplement: Attachment — Submitted filename: rensponse to reviewer.docx [file pgph.0004649.s007.docx]

**Review Comments to the Author**

We sincerely appreciate the time and effort that you and the reviewers have dedicated to evaluating our manuscript. We are grateful for the constructive feedback, which has helped us improve the clarity, accuracy, and overall quality of our work.

We have carefully addressed each of the comments and made the necessary revisions to the manuscript. Below, we provide a detailed response to all the concerns raised, highlighting the specific changes implemented. All modifications are clearly marked in the revised manuscript for ease of reference. We believe these revisions have strengthened our study, and we appreciate the opportunity to refine our work based on the valuable input received. Thank you for considering our submission, and we look forward to your further assessment.

Reviewer #1: (No Response)

Reviewer #2: Abstract
**Comment**
result
Lack of
"adherence to hypertension medication (adjusted odds ratio (AOR): 5.1; 95% CI: 2.41-
9.78; p < 0.001), having hypertension (AOR: 29.7; 95% Cl 2.30-384.1, p=0.009), being
in employment (AOR: 3.80; 95% CI 1.53-9.46) and hospitalization (AOR: 3.48; 95% CI:
1.61-7.49; p<0.001) were positively associated with hypertensive crisis"
this kind of reporting can confused readers. example those with employment were 3.8 times more like to adhere to medication compare to.....................: this will give clear pictures of what you want to report

**Response:** Thank you for your suggestion. We have revised the way the results are reported to enhance clarity as you have suggested.

**Comment**

Conclusion: thee is no need to report , "with a prevalence of 18.9% (189
per 1,000 patients)"this in your abstract conclusion.

**Response:** Thank you. We have removed this from the conclusion as suggested.

methods and materials
Sample size
91 The estimated minimal sample size was 1007. We estimated a prevalence of 6.3 % from
92 a study conducted in Tanzania [4]. The alpha level of 1.5% and design effect of 1. We
93 used [openepi.com](http://openepi.com/) software to calculate the sample size.
show us the formulary that you used in this work. how did you apply the mathematical formula
**Response:** Thank you for your comment. We have now included the formula used to calculate the sample size and have explained how it was applied in our study.
result

Relationship between hypertensive crisis and other covariates. the authors used p-value so the authors should should re-interpret the result again and they should stop using the percentage. example Age (<0.001), employment (0.002) was significantly associated with.....................

**Response:** Thank you for your feedback. We have reinterpreted the results and removed the percentages from the interpretation.
